# First‐trimester blood urea nitrogen and risk of gestational diabetes mellitus

**DOI:** 10.1111/jcmm.14924

**Published:** 2020-01-10

**Authors:** Pei Feng, Guangli Wang, Qian Yu, Wei Zhu, Chongke Zhong

**Affiliations:** ^1^ Kunshan Maternity and Children's Health Care Hospital Kunshan China; ^2^ Department of Epidemiology School of Public Health and Jiangsu Key Laboratory of Preventive and Translational Medicine for Geriatric Diseases Medical College of Soochow University Suzhou China

**Keywords:** blood urea nitrogen, chronic kidney disease, gestational diabetes mellitus, insulin resistance

## Abstract

Prior studies indicated that urea increased insulin resistance and higher blood urea nitrogen (BUN) was associated with incident diabetes mellitus. However, it remains unclear whether BUN during the first trimester of pregnancy increases risk of gestational diabetes mellitus (GDM). We aimed to investigate the association between first‐trimester BUN and risk of incident GDM. We conducted a prospective, multicenter cohort study of pregnant women. A total of 13 448 eligible pregnant women with measured first‐trimester BUN levels were included in this analysis. Logistic regression analysis was used to estimate the relationship between BUN and GDM. Discrimination and reclassification for GDM by BUN were analysed. A total of 2973 (22.1%) women developed GDM. Compared with the lowest quartile of BUN, the third and fourth quartiles were associated with increased risk of GDM (adjusted odds ratios 1.21 [95% CI 1.07‐1.37] and 1.50 [95% CI 1.33‐1.69], respectively, *P* for trend <.001). The addition of BUN to conventional factor model improved discrimination (C statistic 0.2%, *P* = .003) and reclassification (net reclassification index 14.67%, *P* < .001; integrated discrimination improvement 0.12%, *P* < .001) for GDM. In conclusion, higher BUN concentrations during the first trimester of pregnancy were associated with increased risk of GDM, suggesting that BUN could be a potential predictor for GDM.

## 1 INTRODUCTION

Gestational diabetes mellitus (GDM) is defined as glucose intolerance with onset or first recognition during pregnancy.^1^ Women with GDM not only have increased incidence of pre‐eclampsia, macrosomia and caesarean section,^2^ but also experience higher risk of developing type 2 diabetes mellitus after pregnancy.^3^ It is documented that women with GDM had at least a sevenfold increased risk of developing type 2 diabetes mellitus after delivery compared with those who have had a normoglycaemic pregnancy.^4^ Additionally, children with maternal diabetes exposure are more likely to develop diabetes in youth.^5^ Accurate identification and management of risk factors of GDM are highly desirable for optimize care and interventions.

Chronic kidney disease (CKD) has become a worldwide public health problem and is commonly characterized by insulin resistance.^6^, ^7^ It has been shown that CKD and diabetes share similar pathological mechanisms.^8^ Blood urea nitrogen (BUN) is conventionally considered as a parameter to evaluate renal function and had been reported to be associated with cardiovascular events and mortality in various pathophysiological conditions.^9^, ^10^ Prior experimental studies suggested that increased levels of urea might induce insulin resistance and suppress insulin secretion both in vitro and in vivo. Recently, epidemiological evidence from the US Department of Veterans Affairs databases provided the evidence that higher levels of BUN were associated with increased risk of incident diabetes mellitus among people without diabetes^11^ and increased risk of insulin use in patients with diabetes.^12^ However, whether BUN during the first trimester of pregnancy increases risk of incident GDM remains unknown. Hence, we aim to explore the relationship between first‐trimester BUN and GDM in a larger cohort of pregnant women.

## 2 METHODS AND METHODS

### 2.1 Participants

The multicenter cohort study was conducted in ten hospitals and fifteen community healthcare centres of Kunshan, Jiangsu Province, China, from January 2015 to December 2017. The aims of this cohort study were to identify first‐trimester predictors of adverse pregnancy outcome. Pregnant women receiving prenatal care at participating hospitals or healthcare centres were invited to participate in this cohort. Women 14‐40 years old were included, and exclusion criteria were without first‐trimester data of BUN, receiving first prenatal care after 14 weeks, drug and/or alcohol abuse and uncontrolled endocrine disease. Finally, a total of 13 448 women were eligible for this analysis.

The study was approved by the Ethics Committee of Maternal and Child Health Hospital of Kunshan, and written informed consent was obtained from all study participants.

### 2.2 Data collection and outcome definition

Baseline data on maternal demographic characteristics, such as maternal age, gravidity and parity, and educational levels, were collected at the first trimester with a standard questionnaire by a face‐to‐face interview with pregnant women. During each pregnancy visit, healthcare professionals gave some advice and conducted relevant medical examinations including physical examination and electrocardiography based on the health status of pregnant women. Three blood pressure (BP) measurements were also obtained by trained nurses using a standard mercury sphygmomanometer. Body weight and height were measured with participants wearing light clothing and without shoes. Body mass index (BMI) was calculated as weight in kilograms divided by the square of the height in metres. Gestational weight gain was defined as the increase of weight from the first pregnancy visit to the last visit. Blood samples were collected within 24 hours of hospital admission. Laboratory variables, including serum lipids, fasting plasma glucose (FPG), BUN and creatinine, were assayed at local laboratories. Estimated glomerular filtration rate (eGFR) was defined according to the Chronic Kidney Disease Epidemiology Collaboration creatinine equation with adjusted coefficient of 1.1 for the Chinese population.^13^

Gestational diabetes mellitus was defined as any one 75‐g oral glucose tolerance test (OGTT) value ≥5.1 mmol/L at 0 hour, 10.0 mmol/L at 1 hour or 8.5 mmol/L at 2 hours between 24 and 28 gestational weeks according to China's Ministry of Health criteria.^14^

### 2.3 Statistical analyses

Study participants were categorized into 4 groups based on quartile of first‐trimester BUN levels: Q1, <2.40 mmol/L; Q2, ≥2.40 and <2.91 mmol/L; Q3, ≥2.91 and <3.50 mmol/L; and Q4, ≥3.50 mmol/L. Continuous variables were described as mean ± standard deviation or median (interquartile range [IQR]), and categorical data are expressed as frequency (%). The generalized linear regression analysis was used to test for trend across the BUN quartiles for continuous variables, and the Cochran‐Armitage trend chi‐square test was used for categorical variables.

Logistic regression models were used to calculate odds ratios (ORs) and 95% confidence intervals (95% CIs) of GDM for upper quartiles (Q2‐Q4) compared to the lowest quartile (Q1) and for 1‐SD increment of logarithm‐transformed BUN levels. The multivariate‐adjusted models included maternal age, education, gravidity, parity, baseline BMI, gestational weight gain, systolic BP, baseline FPG, white blood cell (WBC), haemoglobin and eGFR. A receiver operating characteristic (ROC) curve was further configured to explore cut‐off point of first‐trimester BUN that optimally predicted GDM. In addition, spline regression models were used to examine the shape of the association between BUN and GDM by fitting a restricted cubic spline function and 4 knots (5th, 35th, 65th and 95th percentiles).^15^ Sensitivity analysis was conducted to test the robustness of our results by excluding participants with low eGFR (<90 mL/min per 1.73 m^2^). In subgroup analyses, we conducted multivariable models to examine effect modification by maternal age, gravidity, baseline BMI, gestational weight gain, systolic BP, WBC and haemoglobin. The interaction between BUN and interested factors was tested by the likelihood ratio test of models with interaction terms.

Furthermore, C statistic, category‐free net reclassification index (NRI) and integrated discrimination improvement (IDI) were calculated to evaluate the improvement of discrimination and reclassification by adding first‐trimester BUN to conventional risk factors model.^16^ Two‐tailed *P* < .05 was considered to be statistically significant. All statistical analyses were conducted by SAS statistical software (version 9.4).

## 3 RESULTS

### 3.1 Baseline characteristics

The present analysis included a total of 13 448 women, mean age of whom was 27.63 ± 4.09 years, and the median serum BUN concentration was 2.91 mmol/L (IQR, 2.40‐3.50 mmol/L). The baseline characteristics are presented in Table 1. Compared with the participants with lower BUN levels, those with higher BUN levels tended to be older, had higher proportion of gravidity (≥2), senior high school, higher levels of gestational weight gain, FPG, haemoglobin, serum creatinine and eGFR, but had lower levels of BMI and diastolic BP, and lower proportion of junior middle school or below.

**Table 1:** Characteristics of participants according to serum blood urea nitrogen quartiles

Characteristics	BUN (mmol/L)				*P*‐trend
	Q1 (<2.40)	Q2 (2.40‐2.91)	Q3 (2.91‐3.50)	Q4 (≥3.50)	
No. of patients	3209 (23.86)	3509 (26.09)	3225 (23.99)	3505 (26.06)	
Maternal age (y)	27.33 ± 4.07	27.67 ± 4.05	27.79 ± 4.13	27.70 ± 4.09	.001
Gravidity					
<2	1188 (37.02)	1257 (35.82)	1072 (33.24)	1199 (34.21)	.007
≥2	2021 (62.98)	2252 (64.18)	2153 (66.76)	2306 (65.79)	
Parity					
No	1007 (31.38)	980 (27.93)	747 (23.16)	647 (18.46)	<.001
Yes	2202 (68.62)	2529 (72.07)	2478 (76.84)	2858 (81.54)	
Education attainment					
Junior middle school or below	552 (17.20)	557 (15.87)	465 (14.42)	457 (13.04)	<.001
Senior high school	2589 (80.68)	2882 (82.13)	2683 (83.19)	2993 (85.39)	<.001
College or higher	68 (2.12)	70 (1.99)	77 (2.39)	55 (1.57)	.225
Baseline BMI, (kg/m^2^)	21.20 ± 2.61	21.06 ± 2.66	20.98 ± 2.61	20.82 ± 2.62	<.001
Gestational weight gain (kg)	14.87 ± 5.39	15.02 ± 5.38	15.01 ± 5.38	15.18 ± 5.46	.027
Systolic BP (mm Hg)	110.85 ± 26.17	109.98 ± 19.92	110.46 ± 10.69	110.65 ± 18.48	.065
Diastolic BP (mm Hg)	70.80 ± 18.20	70.00 ± 7.69	70.07 ± 7.75	70.24 ± 17.73	.017
BUN (mmol/L)	2.10 (1.90‐2.26)	2.65 (2.51‐2.80)	3.19 (3.04‐3.30)	4.00 (3.70‐4.43)	<.001
FPG (mmol/L)	4.61 ± 0.44	4.64 ± 0.46	4.66 ± 0.46	4.65 ± 0.47	.002
WBC (10^9^/L)	7.70 (6.50‐8.90)	7.71 (6.54‐9.04)	7.68 (6.50‐9.00)	7.67 (6.42‐8.91)	.543
Haemoglobin (g/L)	125.50 ± 9.53	125.02 ± 9.96	125.14 ± 9.97	124.60 ± 10.28	.001
Creatinine (mmol/L)	46.00 (40.30‐53.35)	47.00 (41.40‐54.00)	48.70 (42.80‐56.00)	48.60 (41.40‐57.70)	<.001
eGFR (mL/min per 1.73 m^2^)	144.47 ± 16.69	143.41 ± 19.43	141.37 ± 18.16	142.24 ± 26.38	<.001

Note

Data given as mean ± SD, mean (IQR) or n (%).

Abbreviations: BMI, body mass index; BP, blood pressure; BUN, blood urea nitrogen; eGFR, estimated glomerular filtration rate; FPG, fasting plasma glucose; WBC, white blood cell.

### 3.2 Association between BUN and GDM

**Figure 1:**
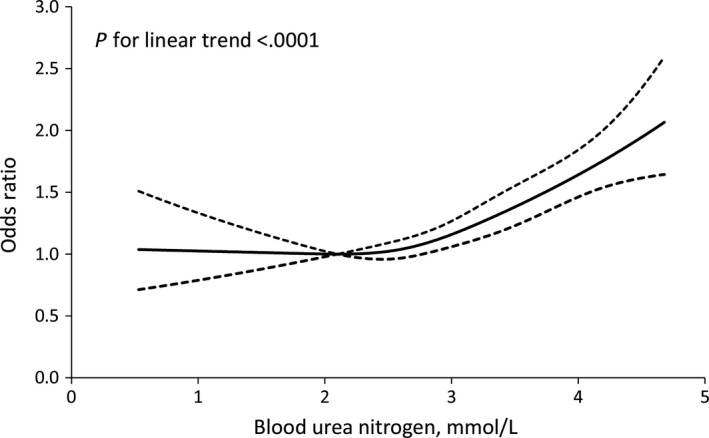
Association of blood urea nitrogen with the development of gestational diabetes mellitus. ORs and 95% confidence intervals derived from restricted cubic spline regression, with knots placed at the 5th, 35th, 65th and 95th percentiles of the distribution of serum blood urea nitrogen. Reference point of serum BUN is the mid‐point (2.10 mmol/L) of the reference group from categorical analysis. ORs were adjusted for maternal age, education, gravidity, parity, baseline BMI, gestational weight gain, systolic BP, FPG, WBC, haemoglobin and eGFR

Among 13 448 women of the analysis, there were 2973 (22.11%) participants who developed GDM. The association between BUN and GDM is shown in Table 2. In unadjusted model, BUN levels were associated with increased risk of GDM, regardless of whether it was treated as a continuous or categorical variable. The association between BUN and GDM was still significant in model 2 adjusting for maternal age, education, gravidity, parity, baseline BMI, gestational weight gain, systolic BP, FPG, WBC and haemoglobin. After further adjusting for eGFR in model 3, the OR for the highest quartile of BUN was 1.50 (95% CI, 1.33‐1.69; *P*‐trend <.001) as compared with the lowest quartile for GDM. An optimal BUN cut point (3.15 mmol/L) was obtained from the ROC curve, compared with the lower group of BUN, and the adjusted OR (95% CI) for the higher group was 1.39 (1.28‐1.51) for GDM. Similarly, on continuous analyses, per 1‐SD increase of logarithm BUN was associated with an 8% (95% CI, 4%‐13%) increased odd of GDM. Multiple‐adjusted spline regression models suggested a linear association between BUN levels and GDM (*P* for linearity <0.001; Figure 1).

**Table 2:** Association between first‐trimester blood urea nitrogen and the development of gestational diabetes mellitus

	N (%)	Model 1		Model 2		Model 3	
		OR (95% CI)	*P*‐value	OR (95% CI)	*P*‐value	OR (95% CI)	*P*‐value
Quartiles							
Q1 (<2.40)	591 (18.42)	1.00 (Ref.)		1.00 (Ref.)		1.00 (Ref.)	
Q2 (2.40‐2.91)	678 (19.32)	1.06 (0.94‐1.20)	.344	1.02 (0.90‐1.15)	.807	1.01 (0.89‐1.15)	.825
Q3 (2.91‐3.50)	745 (23.10)	1.33 (1.18‐1.50)	<.001	1.22 (1.08‐1.38)	.002	1.21 (1.07‐1.37)	.002
Q4 (≥3.50)	959 (27.36)	1.67 (1.49‐1.87)	<.001	1.50 (1.33‐1.69)	<.001	1.50 (1.33‐1.69)	<.001
*P‐*value for trend			<.001		<.001		<.001
Cut‐off of ROC curve							
<3.15	1571 (19.30)	1.00 (Ref.)		1.00 (Ref.)		1.00 (Ref.)	
≥3.15	1402 (26.40)	1.50 (1.38‐1.63)	<.001	1.39 (1.28‐1.51)	<.001	1.39 (1.28‐1.51)	<.001
Per log‐SD increment		1.12 (1.08‐1.16)	<.001	1.08 (1.04‐1.12)	<.001	1.08 (1.04‐1.13)	<.001

Note

Model 1 was unadjusted. Model 2 adjusted for maternal age, education, gravidity, parity, baseline BMI, gestational weight gain, systolic BP, FPG, WBC and haemoglobin. Model 3 adjusted the factors in model 2 and eGFR.

In the sensitivity analyses, further exclusion of participants with low eGFR (<90 mL/min per 1.73 m^2^) did not change the association between BUN and GDM (Table 3). Similar significant associations between high BUN and GDM were observed in all subgroups (Table 4). Statistical tests for interactions between BUN and these interesting factors on outcome were not significant (all *P* > .05).

**Table 3:** Association of blood urea nitrogen and gestational diabetes mellitus: sensitivity analysis

Variables	BUN (mmol/L)				*P*‐trend	Per log‐SD increment
	Q1 (<2.40)	Q2 (2.40‐2.91)	Q3 (2.91‐3.50)	Q4 (≥3.50)		
Excluding participants with low eGFR (<90 mL/min per 1.73 m^2^)						
Cases (%)	583 (18.33)	653 (18.87)	724 (22.75)	927 (26.96)		
Age‐adjusted	1.00 (Ref.)	1.02 (0.90‐1.15)	1.28 (1.13‐1.45)	1.62 (1.44‐1.82)	<.001	1.11 (1.07‐1.16)
Multivariable‐adjusted	1.00 (Ref.)	0.99 (0.87‐1.13)	1.21 (1.06‐1.37)	1.49 (1.32‐1.68)	<.001	1.08 (1.04‐1.13)

Note

Multivariable‐adjusted models were adjusted for maternal age, education, gravidity, parity, baseline BMI, gestational weight gain, systolic BP, FPG, WBC, haemoglobin and eGFR.

**Table 4:** Association of blood urea nitrogen and gestational diabetes mellitus: subgroup analyses

Characteristics	BUN (mmol/L)				*P*‐trend	*P*‐interaction
	Q1 (<2.40)	Q2 (2.40‐2.91)	Q3 (2.91‐3.50)	Q4 (≥3.50)		
Maternal age (y)						
<27 (median)	1.00 (ref.)	0.93 (0.76‐1.13)	1.17 (0.96‐1.43)	1.58 (1.31‐1.90)	<.001	.105
≥27	1.00 (ref.)	1.07 (0.91‐1.26)	1.24 (1.05‐1.45)	1.42 (1.21‐1.66)	<.001	
Gravidity						
<2	1.00 (ref.)	1.05 (0.85‐1.30)	1.11 (0.89‐1.38)	1.40 (1.14‐1.72)	.005	.655
≥2	1.00 (ref.)	1.02 (0.88‐1.17)	1.30 (1.13‐1.50)	1.71 (1.50‐1.95)	<.001	
Baseline BMI (Kg/m^2^)						
<24	1.00 (ref.)	0.99 (0.87‐1.14)	1.22 (1.07‐1.40)	1.45 (1.27‐1.65)	<.001	.495
≥24	1.00 (ref.)	1.10 (0.82‐1.48)	1.13 (0.83‐1.54)	1.72 (1.28‐2.32)	.001	
Gestational weight gain (kg)						
<15	1.00 (ref.)	1.07 (0.90‐1.27)	1.27 (1.07‐1.50)	1.58 (1.34‐1.86)	<.001	.375
≥15	1.00 (ref.)	0.95 (0.79‐1.15)	1.14 (0.94‐1.37)	1.40 (1.17‐1.67)	<.001	
Baseline SBP (mm Hg)						
<110 (median)	1.00 (ref.)	0.99 (0.81‐1.21)	1.18 (0.97‐1.44)	1.48 (1.23‐1.79)	<.001	.511
≥110	1.00 (ref.)	1.03 (0.88‐1.21)	1.22 (1.04‐1.44)	1.50 (1.29‐1.76)	<.001	
WBC						
<7.7 (median)	1.00 (ref.)	0.991 (0.825‐1.191)	1.267 (1.059‐1.515)	1.493 (1.255‐1.776)	<.001	.585
≥7.7	1.00 (ref.)	1.032 (0.869‐1.226)	1.146 (0.963‐1.364)	1.487 (1.259‐1.757)	<.001	
Haemoglobin						
<125 (median)	1.00 (ref.)	1.039 (0.857‐1.258)	1.264 (1.044‐1.530)	1.577 (1.315‐1.892)	<.001	.882
≥125	1.00 (ref.)	0.999 (0.846‐1.178)	1.176 (0.998‐1.386)	1.442 (1.228‐1.692)	<.001	

Note

Models were adjusted for maternal age, education, gravidity, parity, baseline BMI, gestational weight gain, systolic BP, FPG, WBC, haemoglobin and eGFR, except for stratified variables.

### 3.3 Discrimination and reclassification of BUN

Discrimination and reclassification for GDM by BUN are shown in Table 5. Adding BUN to the conventional model significantly improved C statistic by 0.2% (*P* = .003), category‐free NRI by 14.67% (*P* < .001) and IDI by 0.12% (*P* < .001) for GDM.

**Table 5:** Discrimination and reclassification statistics (95% CI) for gestational diabetes mellitus by blood urea nitrogen

	C statistic		NRI (continuous), %		IDI, %	
	Estimate (95% CI)	*P*‐value	Estimate (95% CI)	*P*‐Value	Estimate(95% CI)	*P*‐Value
Conventional model	0.643 (0.635‐0.651)		1.00 (reference)			
Conventional model + log(BUN) (continuous)	0.645 (0.637‐0.653)	.003	14.67 (10.61 to 18.74)	<.001	0.12(0.06 to 0.18)	<.001
Conventional model + BUN (quartiles)	0.650 (0.641‐0.658)	.001	18.03 (13.99 to 22.08)	<.001	0.45 (0.33 to 0.56)	<.001
Conventional model + high BUN (cut‐off)^a^	0.649 (0.641‐0.658)	.001	19.7 (15.66 to 23.74)	<.001	0.46 (0.34 to 0.58)	<.001

Note

Multivariable adjusted for maternal age, education, gravidity, parity, baseline BMI, gestational weight gain, systolic BP, FPG, WBC, haemoglobin and eGFR.

a Optimal cut‐off point obtained from the receiver operating characteristic curve.

## 4 DISCUSSION

In this multicenter cohort study, we observed a dose‐response association between higher first‐trimester BUN and an increased risk of developing GDM, even after adjustment for potential confounders including eGFR. Sensitivity and subgroup analyses further confirmed our findings. To our knowledge, this is the first study to examine the association between BUN during the first trimester of pregnancy and the risk of incident GDM.

A growing body of epidemiological studies have documented that CKD was associated with diabetes mellitus.^17^, ^18^, ^19^ For example, a large cross section of 37 716 female participants from the Kidney Early Evaluation Program (KEEP) found that women with GDM had a higher prevalence of microalbuminuria, even in the absence of subsequent overt diabetes.^18^ Another prospective cohort study conducted in 820 women from Coronary Artery Risk Development in Young Adults (CARDIA) study reported a significant association between GDM and CKD with an adjusted hazard ratio of 1.96 (95% CI, 1.04‐3.67) among black women.^19^ It is likely that diabetes mellitus and kidney disease share similar pathological mechanisms.^8^ On the one hand, diabetes mellitus is a driver of kidney disease,^20^ and kidney disease including urea or other uraemic components may increase the risk of diabetes on the other.^21^, ^22^

Blood urea nitrogen, generally considered as one of the kidney function markers, has been recently reported to be associated with diabetes mellitus.^7^, ^11^, ^23^ However, data from cohort studies on the association of first‐trimester BUN and GDM are scarce. Several experimental studies provided evidence that urea as a putative culprit could increase insulin resistance and suppress insulin secretion.^7^, ^23^ In a previous paper, the capacity of urea synthesis was increased among insulin‐dependent diabetes patients.^24^ Recently, a large cohort of 1 337 452 US Veterans followed for a median of 4.93 years provided epidemiologic evidence to support the finding that a higher concentration of BUN was associated with an increased risk of incident diabetes mellitus.^11^ Our results extended this information specifically to pregnant women population and provided epidemiologic evidence to support the relationship between BUN and developing GDM. In the present study, we found that a higher level of first‐trimester BUN was significantly associated with GDM, and there was a linear dose‐response relationship between BUN and GDM. More importantly, the addition of BUN to conventional risk factors improved risk prediction for GDM, suggesting that first‐trimester BUN could provide important predictive information for the development of GDM.

Although the underlying causes of BUN affecting GDM are still unclear, several potential pathophysiological mechanisms have been proposed to explain the association. The increased concentration of urea induced reactive oxygen species production, which lead to impair insulin signalling through inhibitory serine phosphorylation of insulin receptor substrate.^23^, ^25^ Moreover, experimental studies demonstrated that the disturbance of glucose homeostasis was due to retention of uraemic metabolites including urea, p‐cresyl sulphates, modification of gut microbiome, oxidative stress and inflammation.^7^, ^26^, ^27^ Researchers further observed that beta‐cell dysfunction induced by elevated levels of uraemic metabolite urea is an important contributor to impaired glucose homeostasis in CKD mice.^28^ Furthermore, in human studies, Xie and colleagues recently had found that higher levels of BUN were associated with an increased risk of insulin use among people with diabetes.^12^ Further studies are needed to clarify the mechanisms and to validate our findings in other cohorts.

Our study has some strengths. This is a multicenter prospective study with a large sample size of pregnant women. All data in our study were collected with rigid quality control. Moreover, comprehensive information about potential confounding factors of BUN‐GDM relationship was collected and controlled in the multivariable models. What' more, the sensitivity and subgroup analyses confirmed the significant association between first‐trimester BUN and GDM, suggesting the robustness of our finding. Several limitations should be discussed here. First, although we attempted to control for all relevant potential confounders available in our study, some unmeasured or unknown residual confounders remained due to observational study design. Second, all the participants were from China, so it should be cautious when the findings extrapolate to other populations. Third, our study cannot affirm the association between BUN and overt diabetes after delivery. Further long‐term follow‐up studies are required to clarify the relationship between first‐trimester BUN and diabetes mellitus after delivery.

## 5 CONCLUSIONS

Our results indicated that higher concentrations of BUN during the first trimester of pregnancy were positively and independently associated with increased risk of GDM. Adding BUN to conventional risk factors significantly improved risk prediction for GDM, suggesting that first‐trimester BUN could be a potential predictor for GDM.

## CONFLICT OF INTEREST

None.

## AUTHOR CONTRIBUTIONS

Each author has made an important scientific contribution to this study and has assisted with the drafting or revising of the manuscript.

## Data Availability

The data are free access to available upon request.
